# Uptake of direct oral anticoagulants in primary care: an ecological and economic study

**DOI:** 10.3399/bjgpopen20X101033

**Published:** 2020-05-20

**Authors:** Rachel Denholm, Howard Thom, William Hollingworth, Rupert Payne

**Affiliations:** 1 Centre for Academic Primary Care, Bristol Medical School, University of Bristol, Bristol, UK; 2 Centre for Academic Primary Care, Bristol Medical School, University of Bristol, Bristol, UK

**Keywords:** anticoagulant, warfarin, economics, hospitalization, direct oral anticoagulants, commissioning, randomized controlled trials, primary health care, general practice

## Abstract

**Background:**

Clinical trials indicate that direct oral anticoagulants (DOACs) are as effective as warfarin at preventing ischaemic stroke. It is unclear, however, whether relative changes in DOAC uptake have affected clinical and economic outcomes in practice.

**Aim:**

To investigate variations in DOAC uptake and the relationship with hospital admissions and cost.

**Design & setting:**

An ecological study using electronic administrative records from England, April 2012 to March 2017.

**Method:**

Multivariable regression was used to model practice variation in DOAC prescribing, and the relationship with clinical and economic outcomes.

**Results:**

In quarter 1 of 2017, 55.0% of the 2 695 262 patients dispensed an anticoagulant were given a DOAC. There was a two-fold difference in odds of dispensing DOACs between clinical commissioning groups (CCGs) between those with the highest and lowest usage of these drugs. Increases in the relative uptake of DOACs were not associated with hospital admissions for ischaemic stroke (adjusted incidence rate ratio [IRR] = 1.00; 95% confidence intervals [CI] = 0.999 to 1.001), nor gastrointestinal or intracranial bleeds (IRR = 1.001; 95% CI = 1.000 to 1.002). In 2017, quarter 1, CCGs spent £9247 (inter-quartile range £7751 to £10 853) per 1000 patients on anticoagulants. The marginal effect of a 5% increase in DOAC uptake was associated with a £17.95 (£8.75 to £27.15) increase in total costs, per 1000 patient population.

**Conclusion:**

There were significant differences in the relative uptake of DOACs across practices, with greater costs but no reduction in hospital admissions in those with higher levels of dispensing. Findings indicate that clinical and economic benefits of DOACs identified by clinical trials are not realised in practice.

## How this fits in

Evidence from randomised controlled trials (RCTs) indicate that DOACs are as effective as warfarin at reducing the risk of ischaemic stroke, with greater incremental net benefit. Findings from this national study, the first to investigate the effectiveness and economic costs associated with changes in the relative uptake of DOACs in primary care in England, highlight the variability of DOAC prescribing across general practices. The authors observed no evidence of reduced hospital admissions for ischaemic stroke, and gastrointestinal and intracranial bleeds, associated with greater relative uptake of DOACs, but increases in the cost to CCGs, due to high drug costs which were not offset by a reduction in drug monitoring.

## Introduction

Historically, warfarin has been the dominant preventive treatment for ischaemic stroke for patients with atrial fibrillation (AF) and venous thromboembolism. DOACs have been available in the UK since 2008,^1^ leading to decreases in the prescribing of warfarin in primary care.^2^ While being as clinically effective at reducing thromboembolic events as warfarin, DOACs have the advantage of a rapid onset of action, reduced bleeding risk, and do not require intensive monitoring.^3^

DOACs’ effectiveness has been established by RCTs and observational studies.^4–9^ However, there remain concerns regarding DOACs safety, including managing bleeding complications, increased risk of myocardial infarction,^10^ and concerns over adherence (owing to the lack of monitoring), especially in ‘real world’ settings.^2,11,12^

The cost per patient of DOACs is substantially higher than that of warfarin, but cost-effectiveness analysis has suggested this is offset by reduced therapeutic monitoring, and improved effectiveness and safety.^11^ To date, no economic evaluation has been undertaken using ‘real world’ data, which is needed for health service planning and commissioning.

This study investigated the primary care organisation-level factors associated with increased DOAC dispensing, and whether a shift from warfarin to DOACs is associated with changes to clinical outcomes and costs.

## Method

### Data sources

This retrospective longitudinal ecological study collated national data from April 2012 to March 2017. The NHS Prescription Services’ prescribing databases (ePACT and ePACT2)^13^ contain information on all pharmaceutical products dispensed in English primary care, with ePACT2 (available since 2014) linking products to individual patients. Practice-level information on quantity (that is, number of tablets) and number of patients (ePACT2 only) dispensed anticoagulants, and associated costs, is available monthly for each drug substance (defined by the *British National Formulary* [BNF]).

Data on all English hospital admissions were obtained from Hospital Episode Statistics (HES). Quarterly practice-level demographic information was available from NHS Digital.^14^ Annual clinical and quality of care measures were derived from Quality and Outcomes Framework (QOF) data. Patient-reported quality and continuity of care were determined from the national semi-annual GP Patient Survey (GPPS).^15^ Dispensing data, hospital events and relevant covariates were linked using GP practice code.

### Measurements

Two medication dispensing measures were derived: first, the number of patients dispensed an anticoagulant (ePACT2); and second, the quantity of anticoagulants issued (ePACT) by GPs in England. Anticoagulants included DOACs (dabigatran [*BNF* sub-section 2.8.2.x0], rivaroxaban [2.8.2.y0], apixaban [2.8.2.zo], edoxaban [2.8.2.aa]), and warfarin (2.8.2.v0).

The principal analysis defined admission for ischaemic stroke as a primary HES diagnosis (International Classification of Diseases-10 [ICD-10] code I63^16^) for any care episode within 7 days of admission. Primary diagnoses of intracranial bleeding (ICD-10 codes I160–I62) and gastrointestinal bleeding (K25.0/2/4/6-K28.0/2/4/6, K92.0–2) were included as secondary outcomes. To avoid duplication of incident cases, admissions from consultant clinics or transfers from other hospitals were excluded.

Total cost of anticoagulants was a summation of all anticoagulation medication and related monitoring costs, and acute hospital management costs associated with adverse events (Table S1). Monthly costs for anticoagulants dispensed from each practice were collected from ePACT data. The annual cost of warfarin monitoring was taken from the National Institute for Health and Care Excellence (NICE) clinical guidelines for AF.^17^ The number of patients treated monthly with warfarin was estimated using ePACT2 data and applied to ePACT quantity data. The acute management costs for ischaemic stroke and intracranial bleeds were taken from a cost-effectiveness analysis of oral anticoagulants.^11^ Acute management costs for gastrointestinal bleeding were taken from 2013 to 2014 NHS reference costs.^18^

Rates of ischaemic stroke, and gastrointestinal and intracranial bleeds, and total costs, were calculated per 1000 patient population.

Practice population sizes, used for the denominator in subsequent percentage measures, were obtained from GP registered patient lists and, where missing, QOF data. Numbers of male patients and patients aged ≥65 years, respectively, were ascertained. The ratio of patients aged ≥85 years to those aged 65–84 years was also calculated, with a higher ratio representing a greater number of very elderly patients. CCGs as of 1 April 2018 were used.

Prevalence of AF, coronary heart disease (CHD), chronic kidney disease (CKD), and diabetes mellitus in each practice was collated from QOF data. Total QOF score was used as a surrogate measure of overall practice quality: total QOF points achieved as a percentage of all achievable.

Patients’ overall experience, access to preferred GP, and level of trust was assessed using the GPPS and grouped into binary categories (Supplementary Box S1). Percentage of patients who rated the practice for the aforementioned areas was calculated.

### Statistical analysis

Analysis was conducted at an organisational level. NHS dispensing and hospitalisation data, and total cost, were aggregated to 18 3-month periods. Descriptive analysis investigated variation in exposure and outcome measures across CCGs and over time. Supplementary Box S2 presents further details of the statistical analysis.

### Variation in, and practice-level factors associated with, relative uptake of DOACs (January to March 2017)

To investigate practice-level factors associated with levels of DOAC dispensing between January to March 2017, a mixed-effects logistic regression model with binomial distribution was fitted to calculate odds ratios (ORs) and 95% CIs.^19^ A random-intercept term for CCG was included to allow for differences in dispensing within CCGs, and to explore unmeasured variation in dispensing of DOACs between CCGs.^19^

Univariable analysis was used to investigate the association of DOAC use with individual covariates, before including them together in the full model.

### DOAC uptake, adverse events, and economic outcomes (2012 to 2017)

Multilevel Poisson regression^20^ was used to investigate the number of hospital admissions for ischaemic stroke and bleeds associated with a 5% change in anticoagulants dispensed being a DOAC, with practice included as a random effect to account for clustering. Rate ratios thus represent changes in admission rates when variables within the same practice differ.

Generalised linear models (GLMs) with a Gaussian family and log link function^21,22^ were used to estimate the total costs associated with a 5% change in anticoagulants dispensed being a DOAC. CCG was included as a random effect, and robust standard errors used to allow for potential misspecification of the link and family function.^23,24^

Marginal effects were calculated and represent a change in the outcome for an increase in the exposure, keeping all other covariates at their observed levels, and averaged over all patients. Models were adjusted for demographic, clinical, and practice factors, and non-linear associations for all continuous measures were investigated. Models were restricted to observations with complete information on all variables included in the full model.

All data analysis was conducted using Stata (version 14),^25^ using two-sided significance tests.

### Sensitivity analyses

The robustness of the primary outcome definition was examined by considering primary diagnosis of ischaemic stroke, including diagnoses recorded later than 7 days, compared with the first 7 days of admission only. Relevant secondary diagnoses (in addition to primary), and alternative coding (I64 [unspecified stroke], and G46 [cerebrovascular syndromes] where there is record of cerebral bleed [I60-I62]) were also investigated.

## Results

### Variation in, and practice-level factors associated with, relative uptake of DOACs (January to March 2017)

**Figure 1.**
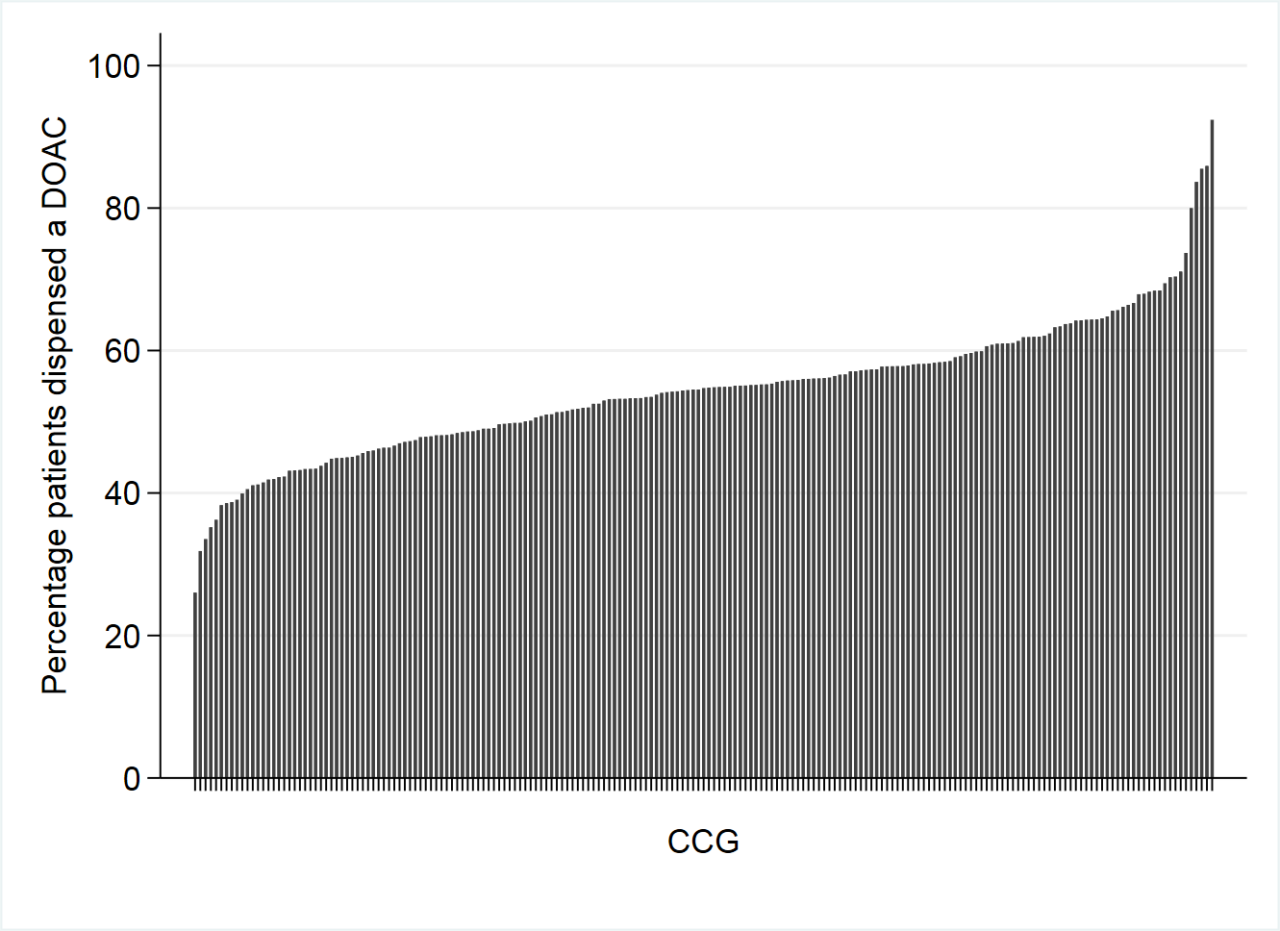
Variation in the relative uptake of DOACs across CCGs, January to March 2017 DOAC = direct oral anticoagulant; CCG = clinical commissioning groups. Anticoagulants include dabigatran, rivaroxaban, apixaban, and edoxaban (DOACs) and warfarin. Percentage of patients dispensed anticoagulants given a DOAC calculation = (Total number of patients dispensed a DOAC/Total number of patients dispensed any anticoagulant) multiplied by 100

Across 195 CCGs, 2 695 262 patients were dispensed anticoagulants, of which 55.0% (*n* = 1 481 638) were dispensed a DOAC. Apixaban was the most frequently dispensed DOAC (26.0% of patients on anticoagulants, *n* = 699 291), and edoxaban the least (0.5%, *n* = 12 689). There was large variation in the relative uptake of DOACs across CCGs (Figure 1), with the lowest in Sutton CCG (26.0% [SD = 8.1%]) and the highest in South Kent Coast CCG (92.4% [SD = 14.6%]).

In the full model, the strongest independent predictor of DOAC uptake was the ratio of ≥85 year olds to 65–84 year olds registered at the practice: OR 1.05 (95% CI = 1.05 to 1.06) per SD (Table 1). Higher diabetes prevalence was associated with lower relative DOAC uptake (OR = 0.94; 95% CI = 0.93 to 0.94), while increasing AF prevalence (OR = 1.02; 95% CI = 1.01 to 1.03) and greater continuity of care (OR =1.01; 95% CI = 1.01 to 1.01) were related to higher relative DOAC uptake. No associations were found for percentage of male patients, patients aged ≥65 years, practice population size, CHD prevalence, or CKD prevalence.

**Table 1.** Association between practice-level factors (per SD) and the relative uptake of DOACs, January to March 2017 (general practices *n* = 6480, CCG *n* = 195)

	**Univariable analysis**	**Full model**
**OR (95% CI**)	***P* value**	**OR (95% CI**)	***P* value**
**Patient demographics**				
% males^a^	0.98 (0.97 to 0.98)	<0.001	1.00 (0.99 to 1.00)	0.708
% aged ≥65 years^a^	0.99 (0.99 to 1.00)	<0.001	0.98 (0.97 to 0.99)	<0.001
Ratio 85:65	1.05 (1.05 to 1.06)	<0.001	1.05 (1.05 to 1.06)	<0.001
Practice population size	1.00 (1.00 to 1.01)	<0.001	1.00 (1.00 to 1.00)	0.915
**Disease prevalence**				
AF	1.00 (1.00 to 1.01)	<0.001	1.02 (1.01 to 1.03)	0.001
CHD	0.99 (0.98 to 0.99)	<0.001	1.00 (0.99 to 1.01)	0.946
CKD	0.99 (0.99 to 0.99)	<0.001	1.00 (1.00 to 1.00)	0.694
DM	0.93 (0.93 to 0.94)	<0.001	0.94 (0.93 to 0.94)	<0.001
**GP** **Patient Survey**				
Experience	1.01 (1.01 to 1.02)	<0.001	1.00 (1.00 to 1.01)	0.628
Preferred GP	1.01 (1.01 to 1.01)	<0.001	1.01 (1.01 to 1.01)	<0.001
Trust	1.01 (1.00 to 1.01)	<0.001	1.00 (0.99 to 1.00)	0.087
Total QOF score^b^	1.00 (1.00 to 1.01)	0.060	1.00 (1.00 to 1.00)	0.771
**CCG variance**				
95% mid-range^c^	1.97 (1.74 to 2.28)	<0.001	1.99 (1.76 to 2.31)	<0.001

Multilevel logistic regression models with a binomial distribution were used, with CCG included as a random effect to account for clustering. All measures were standardised (using sample mean values and SDs), and OR represent the odds of a patient dispensed an anticoagulant (DOAC and warfarin) given a DOAC, for a unit increase in the exposure of interest. DOACs include dabigatran, rivaroxaban, apixaban, and edoxaban

^a^Denominator data used was practice-list size in January–March 2017. ^b^Total QOF score is total QOF points achieved as a percentage of all achievable points (max 559) for 2015–2016. ^c^Calculated from the variance of the random effect (σ2) and is given by e2 ×1.96×σ and represents the odds ratio comparing a practice at the 2.5th percentile of the distribution of practices to one at the 97.5th percentile

AF atrial fibrillation CCG clinical commissioning group CHD coronary heart disease CI confidence intervals CKD chronic kidney disease DM diabetes mellitus DOAC direct oral anticoagulant GP general practitioner HYP hypertension OD odd ratio QOF Quality and Outcomes Framework SD standard deviation

There was variation in the relative uptake of DOACs across CCGs (Table 1). CCGs that dispensed DOACs most frequently (97.5th percentile) were twice as likely to dispense a DOAC, compared with those that dispensed the least (2.5th percentile); OR 1.99 (1.76 to 2.31).

### DOAC uptake and adverse events (2012 to 2017)

**Figure 2.**
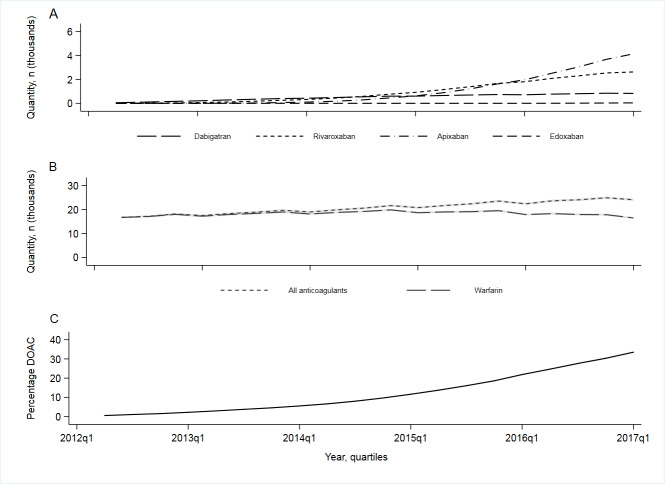
Average (mean) quantity of anticoagulants (DOACs and warfarin) dispensed by a general practice in England, by year quarter. DOAC = direct oral anticoagulant. A: Average (mean) quantity of DOACs (dabigatran, rivaroxaban, apixaban, and edoxaban) dispensed per practice. B: Average (mean) quantity of all anticoagulants and warfarin dispensed per practice. C: Average (mean) relative uptake of DOACs per practice (total quantity of DOACs/total quantity of anticoagulants multiplied by 100) The grey shaded areas represent 95% confidence intervals (mean ±1.96*SD)

The quantity of all anticoagulants dispensed increased over the study period, the most frequently dispensed being warfarin (Figure 2). On average, the quantity of DOACs dispensed per practice rose from 84 prescription items (interquartile range [IQR]: 0 to 300) in 2013, quarter (Q) 1 to 5735 (IQR 2433 to 10 796) in 2017, Q1. The average uptake of DOACs per practice was 31.5% (IQR 23.8% to 39.6%). Since 2016, apixaban has been the most frequently issued DOAC.

**Figure 3.**
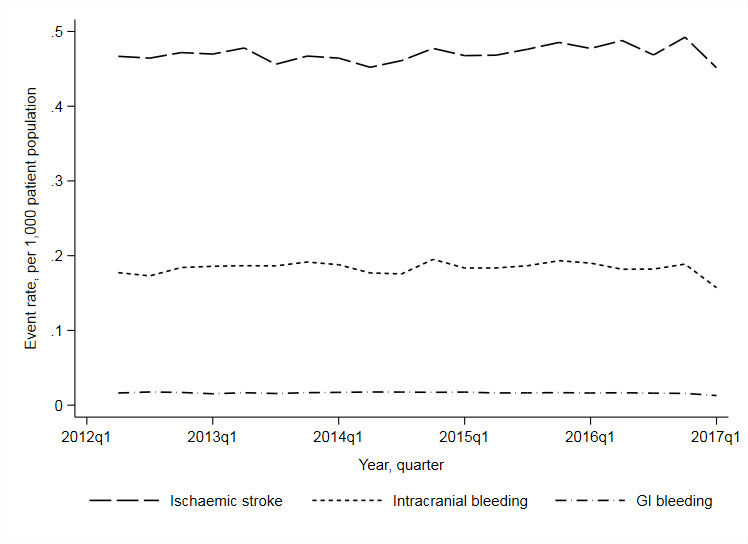
Rates of ischaemic stroke, intracranial bleeding and gastrointestinal bleeding, per 1000 population, by year quarter. GI = gastrointestinal. Ischaemic stroke defined as any I63 ICD-10 code occurring in the primary diagnostic position for any care episode within the first 7 days of admission. Intracranial bleeding (ICD-10 codes I160 to I62) and gastrointestinal bleeding (K25.0/2/4/6-K28.0/2/4/6, K92.0–2) recorded as the primary diagnosis for any episode of care. Denominator data were based on practice population sizes obtained from quarterly reports of patient numbers to NHS Digital, and where missing, annual figures recorded as part of the English primary care pay-for-performance scheme, the Quality and Outcomes Framework.

Rates of ischaemic stroke increased during the study period (incidence rate ratio [IRR] =1.002 (95% CI = 1.001 to 1.003) per 1000 patient population, per quarter), while intracranial and gatrointestinal (GI) bleeds remained stable (Figure 3).

In unadjusted models, there was evidence (*P* = 0.007) that an increase in the relative uptake of DOACs at the practice-level was associated with a decrease in ischaemic stroke admissions (Table S2), although this association was no longer evident after accounting for practice characteristics (IRR = 1.000 [0.999, 1.001] per 5% increase in DOAC uptake). An increase in the relative uptake of DOACs was not associated with changes in hospital admissions from bleeds (intracranial and GI bleeds combined adjusted IRR = 1.001 [1.000, 1.002]).

### DOAC uptake and economic outcomes (2012 to 2017)

**Figure 4.**
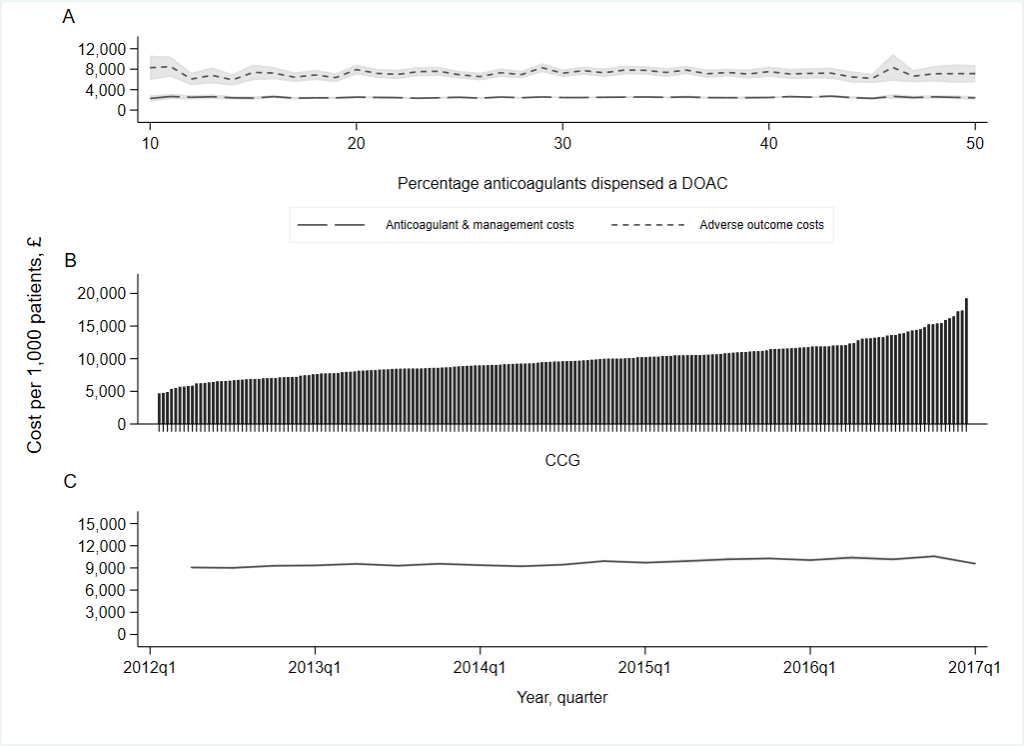
Average (mean) total cost of anticoagulants and associated outcomes, per 1000 patients. DOAC = direct oral anticoagulant; CCG = clinical commissioning group. A: Average (mean) total cost per 1000 patients stratified by percentage anticoagulants a DOAC; January to March 2017. B: Average (mean) total cost per 1000 patients stratified by CCG; January to March 2017. C: Average (mean) total cost per 1000 patients stratified by year quarter. Total cost includes cost of anticoagulants (dabigatran, rivaroxaban, apixaban, edoxaban, and warfarin), management costs of warfarin, and costs associated with a hospital admission for ischaemic stroke, and intracranial and gastrointestinal bleed (see Table S1). The grey shaded areas in Figures A and C represent 95% confidence intervals (mean ±1.96). Figure A restricted to practices within the 95% range of outcome (10%–50% anticoagulants dispensed a DOAC). Analysis excludes practices with practice population <50 (*n* = 1).

In 2017, Q1, the cost of anticoagulants, including monitoring and hospital admission from adverse events, did not change with increases in the relative uptake of DOACs (Figure 4:A). On average, CCGs spent £9247 (IQR £7751 to £10 853) per 1000 patients on anticoagulants in 2017, Q1 (Figure 4: B). Over the study period, the cost of anticoagulants increased by £75.26 (IQR £57.47 to £93.05) per 1000 patient population, per calendar quarter (Figure 4:C), despite a fall in the unit cost of all DOACs (Table S3).

In unadjusted analysis, a 5% increase in DOAC dispensing was associated with a marginal effect of -£16.97 (99% CI = -£27.14 to -£6.81) reduction in total costs, per 1000 patient population. The association attenuated after adjustment, such that increases in DOAC uptake were associated with greater total costs (Table S4); total cost marginal effect £17.95 (£8.75 to £27.15) per 1000 patient population per 5% increase in DOAC uptake (Table 2).

**Table 2.** Marginal effects of number of events and cost per 1000 patients per year quarter, associated with changes in the relative uptake of direct oral anticoagulants

	Number of ischaemic strokes, per 1000 population	Number of bleeds, per 1000 population	Total cost per 1000 population
	Marginal effect (95% CI)	Marginal effect (95% CI)	Marginal effect (99% CI)
per 5% increase	0.000 (0.000 to 0.000)	0.000 (0.000 to 0.000)	£17.95 (£8.75 to £27.15)

CI = confidence intervals. Anticoagulants include dabigatran, rivaroxaban, apixaban, and edoxaban (DOACs) and warfarin. Number of bleeds includes gastrointestinal and cerebral bleeds. Total cost includes cost of anticoagulants (dabigatran, rivaroxaban, apixaban, edoxaban, and warfarin), management costs of warfarin, and costs associated with a hospital admission for ischaemic stroke, and intracranial and gastrointestinal bleed.

**Figure 5.**
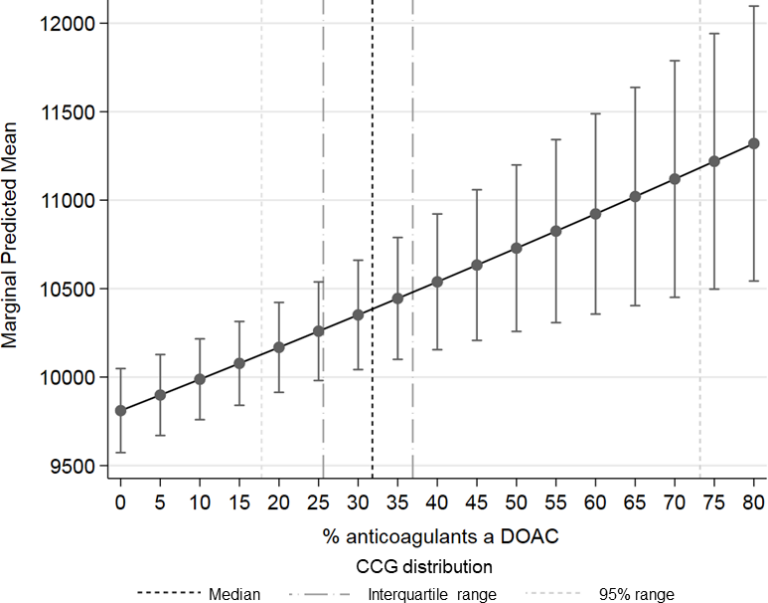
Marginal effect of total cost per 1000 patients, by change in relative uptake of DOACs, with CCG distribution as of January to March, 2017. DOAC = direct oral anticoagulant; CCG = clinical commissioning group. Generalised linear models (GLMs) were used to estimate the costs associated with a change in relative uptake of DOACs, whereby the logarithm of the conditional expectation of the total cost was estimated, and the relationship of the mean to variance in the outcome data were assessed and modelled (models presented in Table S4). CCG was included as a random effect to account for clustering and robust standard errors were used to allow for potential misspecification of the link and family function. Marginal effects represent a change in the outcome for an increase in the exposure, keeping all other covariates at their observed levels, and averaged over all patients. Models were adjusted for demographic, clinical and practice factors CCG distribution reflects the average DOAC uptake in general practices within a CCG. NHS Digital did not have information available before 1 April 2013 so the final models are restricted to after this date.

Across CCGs, those with the lowest practice-average levels of DOAC uptake (2.5th percentile: 18% of all anticoagulants being a DOAC) spent an estimated £10 131 (£9885 to £10 378), per 1000 patient population, compared with £11 179 (£10 479 to £11 880) for those who had the highest (97.5th percentile: 73%) (Figure 5).

### Sensitivity analysis

In sensitivity analysis, changes to the definition and recording of ischaemic stroke, and GI and intracranial bleeds didn’t change the results (data not shown).

## Discussion

This study found substantial variation in the dispensing of DOACs across practices and CCGs in England, with GPs with a higher ratio of older patients and high prevalence of AF more likely to prescribe DOACs. Practices with high levels of diabetes were less likely to prescribe DOACs, potentially reflecting caution in dispensing to some specific patient groups. No association between the relative uptake of DOACs and hospital admissions was found owing to ischaemic stroke or intracranial and GI bleeding, but there were small increases in cost associated with higher rates of DOAC dispensing, which were not offset by the reduction in warfarin monitoring.

### Strengths and limitations

This is the first study to use national dispensing data from England to examine variations in DOAC dispensing across organisations, as well as the impact of relative changes in DOAC usage on hospital admissions and health service costs. It benefits from the use of robust data on practice characteristics and captures virtually all relevant drug dispensing and hospital outcomes.

The principal limitation is the study’s ecological nature, with findings pertaining to practice, not patient, characteristics. Nevertheless, the analysis provides valuable insights into anticoagulant dispensing from a practice and commissioner perspective. The use of longitudinal data with adjustment for clustering by organisation helps reduce some of the limitations of the ecological approach.

### Comparison to existing literature

Previous studies have found warfarin is underutilised for AF, especially among older people.^26^ Increased odds of DOAC dispensing were found in practices with a greater ratio of older patients and those with AF, as shown elsewhere,^2^ indicating DOACs may be preferred by clinicians in this vulnerable patient population.

A systematic review of RCTs found that all DOACS, compared with warfarin, reduced the risk of stroke or systemic embolism (OR = 0.65–0.88 for dabigatran 150 mg twice daily and rivaroxaban 20 mg once daily respectively) and major bleeding (OR = 0.83–0.91 for rivaroxaban 20 mg once daily and dabigatran 110 mg twice daily respectively).^27^ Findings that increased DOAC uptake was equivalent to warfarin in preventing stroke in the general population are consistent with previous analysis of electronic health records data from the UK^2^ and Denmark.^8,28^ The UK study found that apixaban, compared with warfarin, reduced the risk of major bleeding events, in line with RCT findings^2^; the ecological approach, together with grouping all DOACs, may have masked any specific benefit of apixaban in the present study.

This is the first study to examine the direct costs to the health service associated with a shift from warfarin to DOACs, with warfarin costing less owing to the higher unit cost of DOACs in the absence of improvements in outcomes. Previous cost-effectiveness analysis, using RCT data, found a positive expected incremental net benefit of DOACs compared with warfarin.^11^ A recent systematic review of all RCT data found that all DOACs had a higher expected net benefit than warfarin, at a willingness to pay threshold of £20 000 per quality adjusted life year.^29^ This apparent discrepancy may reflect a blunting of DOAC effectiveness in real-world practice, as well as the study’s inability to capture the cost to patients and impact on quality of life. Indeed, evidence indicates that patients receiving DOACs report better quality of life scores and lower levels of depression, compared with patients on warfarin.^30,31^

### Implications for policy and practice

It has been shown that DOAC prescribing has increased significantly across England. Yet despite national guidelines supporting the use of these drugs, underpinned by RCT evidence, there remains considerable variation in dispensing across localities, not explained by differences in patient population. This almost certainly reflects differences and uncertainty in interpretation of the evidence of effectiveness, safety, and cost of these drugs by many clinicians and commissioners. Although certain medications might be more cost-effective in the longer term, commissioners may potentially favour alternative products, which lead to more immediate cost reductions. As costs continue to fall, particularly with patent expiry, DOAC adoption may increase, although this may be tempered if, as advocated by some experts, increased clinical monitoring of these drugs becomes widespread. Furthermore, concerns that beneficial outcomes observed in RCTs may not be borne out in practice are likely to impact on uptake of some new drugs. The study's findings, that DOAC prescribing is more expensive but not offset by clear clinical benefits, help explain why certain organisations appear to be relatively reluctant adopters of these newer products. Both pragmatic RCTs and rigorous analysis of ‘real-world’ data are needed for decisionmaking. However, there is a lack of guidance on how these two sources of evidence should be combined transparently into rational healthcare policy.^32^ Additional work is merited to explore why considerable differences in uptake exist across the country, as understanding these reasons might help inform prescribing recommendations and decisionmaking in the future. Moreover, implementation of new therapies in the real world, involving switching from one drug class to another, such as in this case with warfarin and DOACs, would benefit from being informed by robust policy evaluation of relevant outcomes to ensure that the anticipated benefits are being realised.
