# Correction: Uptake of direct oral anticoagulants in primary care: an ecological and economic study

**DOI:** 10.3399/bjgpopen20X101107

**Published:** 2020-06-15

In the Research by Denholm *et al*. Uptake of direct oral anticoagulants in primary care: an ecological and economic study. *BJGP Open* 2020; DOI: https://doi.org/10.3399/bjgpopen20X101033 , Richard Morris and Sarah Purdy were incorrectly listed as second and third author, respectively. Howard Thom should have been listed as second author and William Hollingworth should have been listed as third author. We apologise for this error. The online version has been corrected.

